# High resolution remote sensing for reducing uncertainties in urban forest carbon offset life cycle assessments

**DOI:** 10.1186/s13021-017-0085-x

**Published:** 2017-10-04

**Authors:** Jan Tigges, Tobia Lakes

**Affiliations:** Geoinformation Science Lab, Department of Geography, Humboldt-Universität zu Berlin, Unter den Linden 6, 10099 Berlin, Germany

**Keywords:** Urban remote sensing, Life cycle assessment, Urban forests, Carbon offset, Uncertainty, High resolution, Individual tree detection, Tree species, Change detection, Climate change mitigation

## Abstract

**Background:**

Urban forests reduce greenhouse gas emissions by storing and sequestering considerable amounts of carbon. However, few studies have considered the local scale of urban forests to effectively evaluate their potential long-term carbon offset. The lack of precise, consistent and up-to-date forest details is challenging for long-term prognoses. Therefore, this review aims to identify uncertainties in urban forest carbon offset assessment and discuss the extent to which such uncertainties can be reduced by recent progress in high resolution remote sensing. We do this by performing an extensive literature review and a case study combining remote sensing and life cycle assessment of urban forest carbon offset in Berlin, Germany.

**Main text:**

Recent progress in high resolution remote sensing and methods is adequate for delivering more precise details on the urban tree canopy, individual tree metrics, species, and age structures compared to conventional land use/cover class approaches. These area-wide consistent details can update life cycle inventories for more precise future prognoses. Additional improvements in classification accuracy can be achieved by a higher number of features derived from remote sensing data of increasing resolution, but first studies on this subject indicated that a smart selection of features already provides sufficient data that avoids redundancies and enables more efficient data processing. Our case study from Berlin could use remotely sensed individual tree species as consistent inventory of a life cycle assessment. However, a lack of growth, mortality and planting data forced us to make assumptions, therefore creating uncertainty in the long-term prognoses. Regarding temporal changes and reliable long-term estimates, more attention is required to detect changes of gradual growth, pruning and abrupt changes in tree planting and mortality. As such, precise long-term urban ecological monitoring using high resolution remote sensing should be intensified, especially due to increasing climate change effects. This is important for calibrating and validating recent prognoses of urban forest carbon offset, which have so far scarcely addressed longer timeframes. Additionally, higher resolution remote sensing of urban forest carbon estimates can improve upscaling approaches, which should be extended to reach a more precise global estimate for the first time.

**Conclusions:**

Urban forest carbon offset can be made more relevant by making more standardized assessments available for science and professional practitioners, and the increasing availability of high resolution remote sensing data and the progress in data processing allows for precisely that.

## Background

Studies around the world have confirmed the role urban forests play in reducing greenhouse gas emissions by storing and sequestering a considerable amount of carbon [1–4]. However, urban forest carbon offset is still regarded with uncertainty due to the lack of precise details on global coverage [5]. Additionally, the urban scale has become more relevant due to the steady failure of global policies to effectively regulate local carbon emissions. Recent carbon ratings and initiatives should therefore not underestimate the local scale of urban forest carbon offset and should be sure to effectively evaluate their potential long-term offset [6].

Urban forests have been discussed as a potential new source of carbon credits, such as by raising revenue for future green investments. Therefore, knowledge of local conditions is essential to better evaluate future opportunities, such as urban carbon offset markets, which have gained considerable interest among local governments [7–9]. This could also take into account aspects of avoided emissions and climate change damage, which are commonly referred to as “social costs of CO_2_ emissions” (SCC) [10]. According to the estimated SCC for 2010 (USD 80 per ton of carbon), US urban forests have stored approximately USD 50 billion in value (2005 level) and have sequestered USD 2 billion in value annually [11]. This creates awareness of the matter by monetizing urban forest carbon offset, but the “real” costs can also be underestimated—for instance, SCC calculations do not necessarily fully consider how climate change affects ecosystems, biodiversity loss and limits of resources, which cause uncertainty in SCC estimates [10, 12].

Urban forest carbon offset is by no means a static value, as its complexity and dynamics need to be considered in order to properly assess its long-term net contribution. An example of a more holistic consideration is a life cycle assessment (LCA) to consider urban forests’ carbon balance throughout a lifetime (cradle-to-grave approach) [13]. This allows for more precisely addressing urban forest heterogeneity and dynamics, for instance: its aspects of structure, planting, maintenance, growth, mortality and more. However, precise information on these features is scarce, not up-to-date or does not cover large spatial expanses, and therefore more standardized methods are required for consistent spatially explicit information [14]. Traditional sampling approaches using land use classes for urban forest carbon estimates are less feasible for precisely addressing in-class variability [15]. There is also no consistent global urban tree canopy model considering precise details [5]. Fortunately, this lack of precise, up-to-date and area-wide urban environmental information can be narrowed using recent innovative technology, such as high resolution remote sensing [16].

The aim of this review is to identify high uncertainties in urban forest carbon offset assessment and discuss the extent to which such uncertainties can be reduced by recent progress in high resolution remote sensing. This would help to improve long-term prognoses of urban forest carbon offset, which is essential for better evaluating the effectiveness of local projects like urban forest carbon offset markets. First, we evaluate state-of-the-art studies on urban forest carbon storage and sequestration. We discuss uncertainties of long-term carbon offset including aspects of supply and demand, and more holistic approaches using LCAs. Second, we identify remote sensing’s potential of high resolution imagery to reduce such uncertainties by providing more consistent and detailed tree inventories. Therefore, we refer to potentials and limitations of classifying urban tree canopies, individual trees and urban tree species. Additionally, we show examples of carbon estimates indicating the benefits of an increasing resolution. In the subsequent section, we provide a case study of urban forest carbon offset for the city of Berlin, Germany. Our case study results and discussion exemplify carbon offset long-term prognoses by combining recent progress of remotely sensed individual tree species and an LCA of 60 years. Finally, we evaluate further remote sensing potentials of increasing temporal resolution to reduce uncertainties related to urban forest dynamics. This includes aspects of urban tree growth, pruning, mortality and replanting.

## Main text

### Urban forest carbon offset—status quo and remaining uncertainties

Quantitative studies on the status quo of urban forest carbon offset are still rare compared to studies on forestry at the national level, but in the 1990s, the availability of urban studies began to spread globally with a focus on US cities. Urban forest carbon storage estimates highly differ between cities with a range from below 10 tC/ha to above 100 tC/ha per unit of tree cover, most of which refers to above-ground carbon storage of a heterogeneous age and species structure regarding a set point in time [1, 4, 11, 15, 17]. In general, forest carbon storage estimates are typically based on well-established allometric biomass equations, with approximately 50% of above-ground dry biomass related to carbon (C) [18]. However, most biomass equations have not been developed specifically for urban trees, resulting in variability in carbon estimates. Furthermore, few studies on urban forests have precisely addressed the heterogeneity and area-wide spatially explicit information. This has subsequently remained a major source of uncertainty and thus requires further assessments [19, 20].

Research on spatiotemporal changes in urban ecosystem services still faces challenges due to non-uniform and multifunctional approaches, different methodologies, diverging stakeholders’ interests or a lack of relevant information [21], among others, information on urban forest carbon offset. Especially the lack of information refers to the dynamics of urban forests, which are associated with natural disturbances like differences in growth, diseases, stresses and natural hazards, or to other anthropogenic disturbances in particular, such as influences of environmental actors, management, political discourses and related land use change dynamics [17, 22, 23]. Climate change effects can also lead to further exacerbations and stress for urban forests [24]. Because of all of this, it is challenging to account for a thorough consideration for future prognoses, such as for urban forest carbon offset in the long-term. However, the consideration of urban ecosystem service dynamics could serve as advice for sustainable future implementation policies [25], or for improved climate change resilience of and through urban ecosystem services [26].

#### Current information on supply and demand

Information on the supply of urban forest carbon storage and sequestration is not commonly available, and information of the demand side is even scarcer. It has been suggested that there is a need to address the supply and demand of such urban ecosystem services in a more spatially explicit manner [27], and environmental quality and policy goals would need to set a standard for the supply and demand [28]. However, it is still arguable just what the minimum standard for this is, though it would be a certain boundary for assessing and managing urban ecosystem services such as urban forest carbon offset. Zhao et al. [29] focused on differences in forest carbon offset (supply) and total carbon emissions (demand) across the Twin Cities (Minneapolis–Saint Paul) metropolitan area of Minnesota, US: sequestration of 2010 could compensate a small rate of 1% of local anthropogenic carbon emissions, as well as the avoided emissions of already stored carbon should not be underestimated. Additionally, there have been obvious spatial differences that have demonstrated that some counties could compensate more than 10% of local carbon emissions using trees, which is slightly higher than what US forests compensate for total US averaged carbon emissions according to information from the Environmental Protection Agency (EPA). Chen [1] showed for 35 major Chinese cities that urban vegetation could offset fossil emission of cities from 0.01% in Hohhot to 22.45% in Haikou in 2010. The dominance of young trees stand of those Chinese cities should not be underestimated, as they will experience substantial growth in the near future. Therefore, forest growth and planting can notably supplement carbon offset in the matter of urban carbon footprinting, which has so far been underestimated. Additionally, the demand of anthropogenic carbon emissions has been dominated by fossil energy up until this point. This offers a great deal of potential for being reduced and substituted by alternatives, which causes a higher contribution of urban forests’ carbon offset. Consequently, this could increase environmental awareness in cities.

#### More holistic approaches using life cycle assessments

An even more holistic perspective on urban forest carbon offset LCA seems to be a promising forward-looking approach for assessing the effects of not just a single stage, but the complete life cycle from creation and use to end-of-life (cradle-to-grave). Until today, few studies have examined urban forest carbon offset using LCA, and most of them addressed select stages concerning planting, growth, maintenance, mortality, reuse of dead biomass or a combination of these qualities. For instance, McPherson et al. [30] showed that, for a one million tree planting initiative for Los Angeles, US, an adequate life cycle management of urban forests can function as a carbon sink to support mitigation goals concerning a 40-year LCA. McHugh et al. [31] also addressed potential tree planting on a citywide scale in Leicester, UK, which showed an as of yet unutilized city area of 15% to increase forest carbon storage concerning a 25-year LCA. Of that area, approximately 50% was also suitable for short-rotation coppice using willow and poplar trees and could therefore act as an additional renewable energy source to replace fossil CO_2_ emissions. Strohbach et al. [32] simulated the carbon footprint (sequestration minus emissions) of selected park trees planted in the city of Leipzig, Germany, with a total net of up to 162 tC/ha for a 50-year LCA (averaging 3.24 tC/ha/year per unit of tree cover) concerning mean tree growth and low annual tree mortality. Although high annual tree mortality (from 0.5 to 4%) could reduce the carbon footprint by over 70%, additional differences in growth rates caused a range of up to 45%. Compared to a citywide scale and simplified tree mortality and decay rates, the urban forests of 28 US cities across 6 states showed a net carbon sequestration rate averaging 2.05 tC/ha/year per unit of tree cover with a range of 0.81 in Minneapolis to 4.01 tC/ha/year in Omaha, demonstrating regional differences in urban forest structure. However, these results are simplified assumptions for the next year and do not account for a more complex long-term LCA [11]. Strohbach et al. [32] showed relatively small emissions—less than 10% of total sequestration—related to construction and maintenance over a long life cycle for tree planting initiatives. However, uncertainty measures on emissions have barely been stated by experts or literature. Emissions due to tree maintenance had been below even 2% concerning the total annual energy consumption of an urban park, as Solà et al. [33] showed in a case study at Montjuïc Park in Barcelona, Spain. However, McPherson et al. [30] addressed the necessity for more efficiency concerning the potential to further avoid emissions during maintenance: irrigation and removal of trees accounted for more than 15% of total CO_2_ emissions, and mulch decomposition of dead tree biomass was responsible for 65.1% during a 40-year LCA of urban trees planted in Los Angeles, US. It also remains unclear the extent to which an increase in maintenance of a single tree and the resulting increase in CO_2_ emissions can lead to extended tree longevity and a positive effect on the net carbon balance [34], or if more efficient maintenance can result in both lower emissions and higher tree longevity.

Concluding the complexity of urban forest carbon offset, LCA seems to be a promising approach for better understanding urban forest dynamics. However, studies on this differ, and most of them address only parts of a complex LCA or consider a small temporal horizon. This has left plenty of space for interpretation regarding how to entirely fulfill the phases of an LCA concerning its suggested formal standard (ISO 14040 and 14044) [13], including: (1) goal and scope definition, (2) inventory analysis, (3) life cycle assessment and (4) interpretation, critical review and reporting. For example, McPherson et al. [30] stated that the available studies on the LCA of urban forest carbon offset have so far failed to account for the full scope of emissions associated at each life stage of a tree. The traditional forestry sector shows similar challenges. Although such studies have been performed since the 1990s, and before urban applications, there is still a lack of consistent and comprehensive studies. Instead, LCA studies differed due to decisive assumptions and their subsequent results concerning system boundaries, functional units, impact categories, considered processes, allocation, LCA methods, available databases, and accuracy metrics [35]. It would certainly be worthwhile to discuss the LCA of urban forest carbon offset in the matter of harmonization or better comparability, such as by increasing precision and data consistency. This should reduce the high variability of LCA results, and especially address differences in tree mortality and growth; forest structures concerning size, age, tree species; data on private and public areas; related data retrieval; (re)planting; actual and potential reuse of dead biomass; and other aspects. It has been challenging to retrieve such necessary data, especially due to the heterogeneous urban forest structure, as well as get access to details across public and private space or beyond administrative boundaries that are also consistent and up-to-date [17, 36, 37]. Improvements should definitely aim at reducing the expensive and time consuming data collection process, as it is an important part of LCAs [13].

### Reducing uncertainties part 1—recent high resolution remote sensing for more consistent urban tree inventories

The development and contributions of high resolution remote sensing have been successfully applied to provide input for improved modeling of urban ecosystem services concerning area-wide details, which are consistent and up-to-date. Generally, this can help to increase the comparability of different sites [38, 39]. Increasing spatial, spectral and temporal resolutions have successfully shown an increase in precision, for example, to monitor the changes at the individual tree level or to identify the diversity of urban tree species [40, 41]. Moreover, unmanned aerial vehicles (UAVs) have expanded the possibilities of urban vegetation mapping and field surveys, and have thus contributed to the growing data availability of remotely sensed imagery [42]. Hence, recent remote sensing offers new monitoring options for dynamic urban environments, for example as a consistent input for urban forest LCAs. High resolution remote sensing has been recommended for supplying more adequate data on local and regional differences and changes in urban forest cover [18], and as a promising standard inventory procedure for assessing the status quo of urban forests [39, 43, 44]. The benefits of high resolution remote sensing data have already been shown by several studies on urban forest carbon offset [11, 45]. However, few studies have identified the extent to which an increasing resolution of remotely sensed data can reduce uncertainties in mapping carbon estimates of urban forests [46, 47]. This also refers to precise remotely sensed data retrieval on urban tree canopies, individual trees and species, which are of utmost importance for an LCA.

#### Urban tree canopy classification

The urban tree canopy is most often used as a baseline parameter for carbon estimates. Urban forests have been classified from remotely sensed data with a threshold of vegetation indices like NDVI (normalized difference vegetation index), which considers the sensitivity of spectral bands near-infrared and red. Combined thresholds of remotely sensed height data have commonly been used to classify the urban tree canopy [48]. However, the absence of standardized metrics make it difficult to compare available urban carbon studies; for instance, the combination of data sets with different acquisition times causes inconsistency [39]. Uncertainty in urban tree canopy classification is often not reported, or is stated with a bias [49]. Despite very high resolution data, Raciti et al. [47] accounted for 14% of the variability in class accuracy occurring by chance when mapping the urban tree canopy using airborne LiDAR height data (1 height point/m^2^) and QuickBird satellite data (2.4 m pixel size). Similar errors were stated in other very high resolution studies. They occurred with objects that were smaller than a remotely sensed pixel (mixed pixel problem), which mostly appeared for urban vegetation with high proximity to the edges of buildings [50]. As this situation is frequently found in densely built areas, it is likely to bias spatially explicit carbon estimates in the end. The quantitative effects of such very high resolution problems have rarely been considered as of yet. This can be overcome by further increasing the resolution in the centimeter range, but at the cost of extensive resource requirements, especially for processing [51]. Therefore, we suggest a minimum quality of urban tree canopy mapping because it is a standard baseline parameter of forest carbon offset and will have a significant influence within an LCA.

#### Individual tree detection

Individual tree information, such as tree growth and mortality issues, is highly relevant for urban carbon estimates if we want to address variability within land cover/use classes. However, the acquisition of urban tree inventory data is resource intensive and still lacks systematic monitoring [52]. This has made remotely sensed individual tree detection (ITD) a great advantage, though it has so far only rarely been applied in the urban context [53]. The ITD for urban forest carbon estimates has minimal scaling effects compared to traditional accurate sampling approaches of land use classes, which instead address a coarse neighborhood or city level [47, 54]. Therefore, uncertainty of carbon estimates is likely to be reduced by remotely sensed ITD, which delivers more precise information regarding differences within and between land cover/use classes. Additionally, remotely sensed ITD is independent from administrative boundaries and offers possibilities of spatial expansion concerning more precise information of the urban–rural gradient.

Early ITD approaches have made use of multispectral remote sensing data for classifying overstory tree crowns [55]. The feasibility of ITD for estimating tree dimensions and biomass has grown alongside the availability of high resolution LiDAR data as three-dimensional point clouds (echo-based), the very high point densities of which can penetrate the tree canopy and subsequently improve understory estimates [56]. Alternatives are the frequently available processed height products, such as canopy height models (CHM), most of which are simplified discrete return LiDAR data reduced to the first return signal (upper tree canopy) [53]. Additional adequate height data for ITD has been offered by photogrammetry options of very high resolution multispectral sensors [57], which has gained attention due to the ever decreasing equipment costs of UAVs offering accuracy at cm-level resolution [58, 59].

Kaartinen et al. [60] provided a comprehensive review of tree detection methods based on the international benchmarking study “Tree Extraction.” The results of a heterogeneous forest test site were highly affected by the chosen algorithm (range between 40 and 90% of trees correctly detected) rather than a CHM of different point density (2–8 points/m^2^). This showed that local maxima finding algorithms provides fast and ready to use results, as well as advanced algorithms like minimum curvature-based tree detection to deliver precise estimates for a tree inventory. The tree height reached an RMSE of 0.5 m. Automated methods also outperformed manual tree detection from remotely sensed data, which implies that field data can be more relevant as a reference for validating and correcting derived estimates. Compared to very high point density LiDAR data, Orka et al. [61] showed fairly accurate estimates of tree height (RMSE, 0.76–0.84 m) and derived stem diameter (RMSE, 3.10 to 3.17 cm) for a highly heterogeneous non-urban forest structure (echo-based LiDAR point clouds, small footprint < 21 cm, a mean pulse density of about 5/m^2^ with a total return of 30–100 echoes). The ITD of very small and young trees could benefit from a higher point density (> 10 points/m^2^) [60]. Additionally, the results of original LiDAR point clouds seemed to output more realistic forest structures than CHM-based ITD [62]. Higher variability of CHM-based results could also be due to smoothing algorithms, interpolation methods and chosen grid spacing during model production [53].

Unfortunately, the above-mentioned ITD approaches have not yet been developed specifically for urban forests. The heterogeneous urban forest structure is extremely challenging due to a mixture of spatial arrangements and a high diversity of tree species, height and age structure. Lee et al. [63] accounted for a mixture of 20 predominant urban tree species using LiDAR data (4 returns per pulse in addition to an LiDAR intensity value). The results provided fairly accurate estimates of tree height (RMSE, 1.64 m) and derived stem diameter (DBH) (RMSE, 10.32 cm). Lee et al. suggested an adequate validation of different tree sizes and groupings to correct potential over- or underestimates. Zhang et al. [53] developed a new algorithm for automated urban tree detection using original echo-based LiDAR point clouds (3.5 points/m^2^) with promising results (R^2^ above 0.84, RMSE 0.57 m for tree height, RMSE 1.9 m for crown diameter). They adapted a local maxima and constrained tree climbing algorithm to detect tree peaks by incorporating a horizontal threshold to better address less pointed forms of deciduous tree species. They also felt that a minimum height threshold was appropriate in order to exclude shrubs. The outer boundaries of individual trees were segmented using an inverse tree climbing method referred to as “the donut expanding and sliding method.” The results should be improved regarding more complex crown shapes, as individual trees were assumed to have a circular shape. For example, Ardila et al. [41] successfully used an automated region-based active contours approach for multispectral data, which allowed for better addressing realistic crown shapes and could better handle the spectral variability of neighboring pixels, which has been a significant challenge for very high spatial resolution imagery. This active contours approach might be improved if extended by photogrammetric three-dimensional data of multispectral imagery. An object-based segmentation approach could also be used to reduce the spectral variability of remotely sensed data [64], but this is more labor intensive and sensitive to the expert’s classification rules [41]. Additionally, algorithms for tree segmentation would have to consider potential over-segmentation of individual tree crowns, especially due to very high resolution data [65]. Recent progress in low-altitude UAV photogrammetry could also provide precise terrain models to reduce the effect of heterogeneous vegetation cover [66], as the lack of adequate ground points can also lead to significantly underestimating urban tree dimensions [67]. Original full waveform LiDAR, which samples a returned signal at the LiDAR sensor with a very high rate appearing as a wave, or synergies combining different sensors using higher spatial, spectral and temporal resolution, could further increase the precision of urban tree dimensions [53, 68–72].

The increasing resolution of remotely sensed imagery and the development of methods and carrier systems are likely to supplement traditional urban field surveys if no up-to-date data is available or there is no data at all. However, a comprehensive review published by Zhen et al. [72] pointed out that ITD methods and data would require more suited applications, such as for urban conditions, and that assessment metrics are inconsistent for evaluating and comparing results. More than 200 studies from 1990 to 2015 were published on ITD, and applications in urban settings have increased during the last 10 years. In the near future, the use of low-cost UAVs is likely to increase as it offers multiple options. For instance, Jaakkola et al. [73] attached a GPS/IMU positioning system, two laser scanners, a CCD camera, a spectrometer and a thermal camera to UAVs. This setup allowed for classifying individual tree heights with a standard deviation of 30 cm for a heterogeneous urban forest.

#### Urban tree species classification

The composition of tree species becomes relevant for a heterogeneous forest structure particularly, for instance, concerning stress resistance, species and site-specific differences in growth and mortality, or differences across large areas. Therefore, numerous studies have addressed tree species classification using high resolution remote sensing.

Recent Worldview 2 multispectral satellite imagery from late spring (May, 8 spectral bands, 2 m pixel size) could improve the classification of seven urban tree species in Tampa, Florida, US using a higher number of spectral bands [74]. For example, the overall accuracy could be increased by approximately 5% compared to IKONOS satellite imagery of early spring with less spectral information (April, 4 spectral bands, 4 m pixel size). However, additional spectral information of Worldview 2 did not exceed an overall accuracy of 63%. Immitzer et al. [75] classified 10 tree species with a good overall data accuracy of 82% in a temperate heterogeneous structured non-urban forest in Austria using Worldview 2 satellite imagery of high summer (July, 8 spectral bands, 2 m pixel size). However, species-specific results highly varied and could drop by 40–60%. Whereas a higher temporal resolution of satellite imagery could improve the classification of urban tree species, such as seasonal data of RapidEye satellite imagery (5 spectral bands, 6.5 m pixel size), which correlates to differences in vegetation phenology. Such multitemporal data could significantly decrease the allocation problem between eight dominant tree species in Berlin, Germany, whereas a single image did not provide sufficient information for classifying tree species. Furthermore, only a few selected spectral information from a multitemporal feature space were relevant for reaching high classification accuracy [40]. The benefits of multitemporal satellite imagery reflecting seasonal differences were confirmed by Li et al. [76], who applied WorldView 2 and 3 imagery of late summer and high autumn to classify urban tree species with satisfying results at two study sites in Beijing, China (overall accuracy > 80 and 90%). More recent studies of very high temporal resolution could further improve tree species classification based on phenological trajectories: Sheeren et al. [77] could classify 13 major tree species in temperate forests in southwest France with a very high degree of accuracy (κ values above 0.95) using a Formosat-2 satellite time series (4 spectral bands, 8 m pixel size) on 17 dates across the year of 2013; Karasiak et al. [78] could perform similar results for the same study site using a Sentinel-2 satellite time series (10 spectral bands, 10 m pixel size) on 11 dates from winter 2015 to autumn 2016. Hyperspectral remote sensing offers a very high number of bands with precise spectral information, which showed fairly accurate results for tree species classification [79, 80]. Structural features derived by high point density LiDAR data allowed similar overall accuracies above 75% within a heterogeneous non-urban forest [81]. Original full waveform LiDAR could further improve the overall accuracy compared to approaches using traditional preprocessed LiDAR data, which is mostly decomposed to discrete peak data in particular [82]. The intensity data recorded for each laser point in a LiDAR system also slightly improved species classification [83]. Furthermore, the combination of sensors can be promising for improved classifications. For instance, combining hyperspectral and LiDAR in particular increased the accuracy for specific tree species from 29% to 71% (tall palm species, *Washingtonia robusta*) [84]. Recent progress in three-dimensional hyperspectral remote sensing should receive more attention in the future, which Nevalainen et al. [85] applied for ITD (number and location) and species classification in boreal forests using a combination of a low-altitude UAV-based photogrammetric point cloud for CHM model production (10 cm raster) and hyperspectral imaging (33 spectral bands, 10 cm ground sampling distance). Nevalainen et al. applied a Random Forest classifier for tree species and reached an overall accuracy of 95%, but varied between 40 and 95% for detecting individual trees due to the heterogeneous site conditions using a local maxima filter algorithm integrated into Fusion software by McGaughey [86]. In this context, we suggest comparing the accuracy and precision of further available ITD approaches.

Recent available studies clearly indicate a high potential for classifying tree species under heterogeneous conditions using high resolution remote sensing. Fassnacht et al. [87] reviewed currently available tree species classification studies and identified promising local scale approaches with multiple options of available remote sensing data. However, recent results still lack adequate transferability across large areas. Experts requested a better understanding of tree species differentiation concerning structural, spectral or phenological indicators rather than solely data-driven statistics, most of which are highly locally tailored classification achievements. To the authors’ knowledge, an approach does not yet exist that solely addresses a single tree species. This would be highly appropriate concerning the prevalence of other native and alien species within urban forests, which is still an information gap [88]. More specifically, future research will need to discover whether there is a unique indicator or a combination for a single tree species using remote sensing data, or if we are limited to distinguishing between species using a more locally tailored approach. This should not be taken to underestimate recent options for making use of remotely sensed tree species classification. However, approaches should be adapted to the selected location and at least include the area’s most dominant tree species, therefore employing a more representative tree population. This would contribute to better assessing the overall classification accuracy of tree species.

#### Examples concerning the effects of an increasing resolution on carbon estimates

Urban forest carbon estimates at different scales exemplify the importance of an increasing resolution for spatially explicit information. The local carbon estimates (individual tree level) of Boston, US were highly underestimated by available national datasets of the same area by 30% (NBCD, National Biomass and Carbon Dataset, 30 m pixel size) to more than 90% (United States Department of Agriculture Forest Service Forest Inventory and Analysis, USDA FS-FIA, 250 m pixel size) [47]. Less uncertainty for increasing resolution has been confirmed for urban forest carbon estimates at the neighborhood level by up to 11% (LiDAR point density 1.2–5.8 points/m^2^) [46]. Singh et al. [89] addressed the challenges of decreasing resolution from a maximum of ca. 14–0.15 points/m^2^ for regional carbon estimates of urban forests in Charlotte, North Carolina, US because processing very high point density LiDAR data is resource intensive and is likely to hold redundant information. Just 40% of the available LiDAR points were sufficient without compromising the accuracy of biomass estimates. This was also confirmed by Garcia et al. [56] for national forest carbon estimates, who found that a reduction of LiDAR point density could be sufficient (10–5 points/m^2^) except for very low point density. It remains unclear for urban forest carbon estimates if metrics derived from processed discrete returns of a crown height model show a higher dependence on point density than metrics derived from the original echo-based LiDAR data as they did for forests in the study by Garcia et al. [56].

Regarding a national scale of urban forest carbon offset, Nowak et al. [18] set a standardized urban forest carbon value from selected cities and upscaled it across the US using national tree canopy data retrieved from 1991 remote sensing imagery of advanced very high resolution radiometer (AVHRR). Due to its coarse spatial resolution of 1 km pixel size, higher resolution Landsat TM satellite imagery of 28.5 m pixel size was used for defined regions to determine their proportion of tree canopy within a coarse AVHRR pixel, which correlates to the magnitude of the spectral response. Such an unmixing approach could then be used to determine the tree canopy’s density within other AVHHR pixels [90]. Nowak et al. [11] updated this approach in 2002 due to an increased availability of better tree cover estimates derived from high resolution remote sensing imagery, which led to a correction of the national average of urban forest carbon density (2002: 92.5 tC/ha per unit of tree cover; corrected 2013: 76.9 tC/ha per unit of tree cover). Pasher et al. [91] assessed Canada’s urban forests at the national scale, with carbon storage and sequestration data adapted from US urban forests by Nowak et al. [11]: they selected administrative boundaries (reconciliation units) of dominant urban land use. These selected areas were divided by a 1 km grid. A random selection of grid cells was used for each reconciliation unit to classify the urban tree canopy using a point sampling approach. Samples were manually classified using very high resolution remote sensing orthophotos (10–25 cm pixel size) and upscaled for the total urbanized area of each associated reconciliation unit, which resulted in a final carbon estimate. This improved assessment of the urban tree canopy showed a sequestration rate of 937,000 tC/year for urban forests in Canada, which is 20 times higher than the current official report by Environment Canada (46,000 tC/year), and could be explained by its assumed very conservative urban tree density of 10 trees/ha [91]. Due to the extensive requirements of resources and manual workload, Pasher et al. suggested (semi) automated processing of remote sensing imagery to advance their approach for assessing the urban tree canopy or to provide general rapid re-assessments. They referred to medium resolution satellite imagery (20–30 m pixel size), which is widely available at the national scale and can improve the upscaling process of regional estimates.

Both Nowak et al. [11] and Pasher et al. [91] were able to update national urban forest carbon storage and sequestration estimates, especially concerning higher resolution remote sensing data for a precise urban tree canopy. Their applied methodologies of estimated urban forest carbon removals of Canada and the US are consistent with IPCC [92] standards and can therefore be used for annual inventory reporting to the United Nations Framework Convention for Climate Change (UNFCCC). As such, this could be a first step towards a consistent global approach for urban forest carbon offset and related dynamics. Unfortunately, the individual tree level is too resource intensive to be applied globally as of yet, though a global urban tree density map would highly advance carbon estimates for an LCA equivalent to a recent available global tree density map of 1 km^2^ resolution by Crowther et al. [93]. In this context, continuous evaluation of recent satellite missions of extensive area coverage could further improve global urban forest estimates, such as RapidEye (6.5 m pixel size, 5 spectral bands, daily revisit), TerraSAR X Tandem (e.g. global digital surface model, 12 m pixel spacing) or Sentinel-2 (down to 10 m pixel size, 13 spectral bands) [94–96].

### The Berlin case of urban forest carbon offset—an example of combining remote sensing and LCA

To relate our findings of this review to a more concrete situation, we conducted a simplified LCA of urban forest carbon offset for Berlin, the capital of Germany. The aim of this case study is to assist scientists and professional practitioners to provide basic long-term prognoses for environmental awareness and to utilize the recent progress made in remote sensing, such as providing a more consistent tree inventory. We pointed out the challenges of assumed tree growth, mortality and planting to achieve the sensitization for the role of urban forest carbon offset.

#### Case input data—LCA inventory of remotely sensed individual tree species

**Fig. 1:**
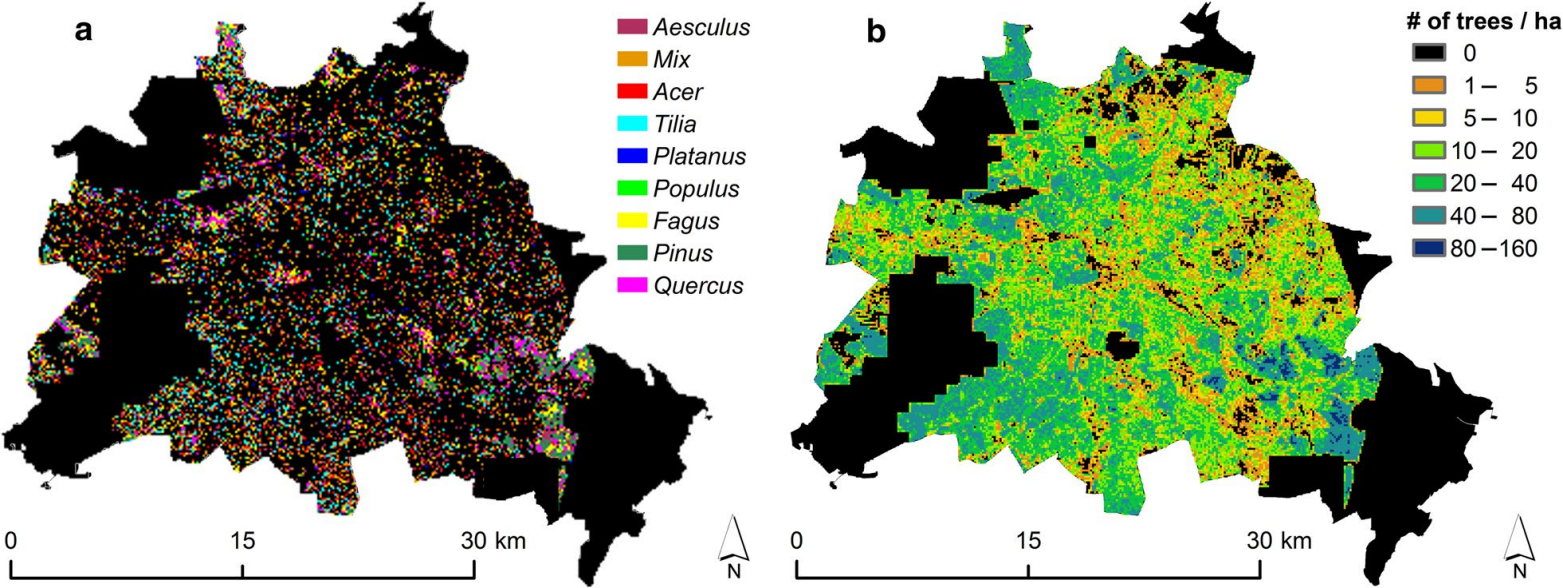
LCA inventory of remotely sensed trees in Berlin, Germany. Spatial distribution of **a** dominant tree species and **b** tree density per unit of land cover [15]. Class “mix” refers to difficult-to-classify tree species in the tree canopy. Approximately 1.4 million trees were classified with a mean tree height of 15 m and a mean DBH of 36 cm. No data was available for the outer black areas, which are mostly covered by forests

The city of Berlin (52°31′N, 13°24′E) has a moderate climate and is characterized by a mostly flat topography. Its administrative area is approximately 890 km^2^, 40% of which is covered by vegetation such as urban forests, parks, street trees and urban agriculture [97]. More than 290 km^2^ of Berlin is taken up by urban forests, which constitutes the largest urban forest in Germany; Berlin’s public parks cover approximately 55 km^2^. Because a consistent and up-to-date tree database covering private and public areas does not exist, we updated our LCA tree inventory using a high resolution remote sensing approach using the final results of individual tree species information, previously published by Tigges et al. [15, 40]: the final tree database contained consistent information concerning location, height, crown width and DBH for each individual tree as well as its species and species fraction of the total tree population. The tree species (Fig. 1) had been derived by applying advanced machine learning on a time series of RapidEye satellite imagery reflecting phenological differences between tree species (5 images during the growth season of 2009; pixel size of 6.5 m). Individual tree metrics were derived from a LiDAR-based tree crown model (winter 2007/2008; regular grid of 4 height points/m^2^), a local maxima filter algorithm that is integrated into Fusion software [86] and laser scanning calculations for DBH estimates from Zhao et al. [98]. Remotely sensed data covered an overlapping area of approximately 700 km^2^ of the city of Berlin, which resulted in a classified urban tree canopy of 213 km^2^ (Fig. 1).

#### Case approach—LCA concerning 60 years of growth, mortality and replanting

Our tree inventory data was entered for an LCA of urban forest carbon offset. The carbon accounting referred to alive and dead above-ground tree biomass and followed a time period of 60 years. We then discussed the role of alive and dead biomass. The initial year of our LCA was set to 2008, according to the date of the LiDAR data. Results were presented at regular 10-year intervals. We accomplished the LCA stages of growth, mortality and planting as follows:

Growth: we used allometric biomass equations and DBH-growth functions (cm/year) for each tree class listed in Table 1 to receive future estimates on tree size and biomass: most dominant individual tree species in the study area are listed as classes 1–10; classes 1–8 are classified using RapidEye satellite data with its fraction of the total tree canopy (CHM); Class “mix” includes all other species of the total tree canopy, mostly comprising classes 9 and 10. Strohbach et al. [32] applied growth data from a street-tree database of Leipzig, Germany, which was utilized for this case study, as non-linear regression functions better reflect a more natural pattern concerning a smaller annual DBH-growth for mature trees (Table 1). We used those functions to convert DBH estimates from our remotely sensed data from 2008 to age estimates of each individual tree. We then simulated a 60-year life cycle for annual estimates of DBH using our adapted growth functions. The results of each year were used as input for allometric equations of above-ground biomass approximations (Table 1). We multiplied our biomass estimates by 0.5 to convert above-ground dry biomass into units of carbon (C) [18]. Because allometric equations demonstrate high under- and overestimates, recent studies require them to be adapted for use in specific urban applications [19]. However, we did not have any specific information for Berlin. Therefore, we carefully selected the species that best matched our case study (Table 1) and did not apply any correction factor for urban biomass estimates as Nowak et al. [18] had done. If no species-specific equations were available, we followed the approach taken by Hutyra et al. [37] by selecting equations of the same genus. If we could not classify a tree species (Class mix, Table 1), we used the average estimate of dominant tree species in our case study area. Our results summarized the total alive biomass for each land use class at 10-year intervals.

**Table 1:** Most dominant tree species for biomass and growth calculations

Class	Genera Tree species	CHM (%)	Biomass estimates Allometric equation	Growth function (DBH); residual error
1	*Acer campestre, Acer platanoides, Acer* sp.	12.9	Equation 2, *Acer saccharum* [99]	$202.9173*(1 - e^{{\left( {age\,*\, -\,0.0019} \right)}} )^{0.7992}$; 5.48
2	*Aesculus hypocastanum*	13.4	Table 1, *Aesculus indica* [100]	$175.5828*(1 - e^{{\left( {age \,*\, -\, 0.0042} \right)}} )^{0.8958}$; 6.57
3	*Fagus sylvatica*	10.4	Appendix A, Equation 89, *Fagus sylvatica* [101]	$202.9173*(1 - e^{{\left( {age \,*\, -\, 0.0019} \right)}} )^{0.7992}$; 6.15
4	*Pinus sylvestris*	11.3	Table 3, *Pinus sylvestris* [102]	$135.4549*e^{{\left( { - \,3.1143 \,*\, e^{{\left( {age \,*\, -\, 0.0152} \right)}} } \right)}}$; 4.74
5	*Platanus hispanica*	1.9	Volume of *Platanus acerifolia*, gravity of *Platanus* [103, 104]	$170.5888*(1 - e^{{\left( {age \,*\, -\, 0.0047} \right)}} )^{0.913}$; 4.95
6	*Populus nigra, Populus alba*	1.8	*Populus tremula* [101]	$93.5402*(1 - e^{{\left( {age \,*\, -\, 0.0152} \right)}} )^{1.1518}$; 10.77
7	*Quercus robur, Quercus rubra, Quercus* sp.	8.6	Table 3, *Quercus* sp. [102]	$70.2797*e^{{\left( { -\, 2.8528 \,*\, e^{{\left( {age \,*\, -\, 0.0289} \right)}} } \right)}}$; 4.03
8	*Tilia cordata, Tilia* × *vulgaris, Tilia platyphyllos*	13.2	Appendix A, Equation 607, *Tilia cordata* [52, 101]	$56.4678*(1 - e^{{\left( {age \,*\, -\, 0.0182} \right)}} )^{1.053}$; 5.71
9	*Betula pendula*	/	Appendix A, Equation 31, *Betula pendula* [101]	$199.1001*(1 - e^{{\left( {age \,*\, -\, 0.0029} \right)}} )^{0.8865}$; 4.48
10	*Robinia pseudoacacia*	/	Table 2, Equation 6, *Robinia pseudoacacia* [105]	$116.6451*e^{{\left( { - \,3.1542\, *\, e^{{\left( {age \,*\, -\, 0.0198} \right)}} } \right)}}$; 5.40
Mix	Mix of dominant species above	26.5	Average of equations listed above	Average of equations listed above

Mortality: estimates of urban tree mortality are affected by high uncertainty due to differences in tree physiology, biophysical and environmental conditions, planting strategies, social-ecological behavior, future climate change and more [30, 106–108]. Due to high uncertainty and simplification we assumed annual static mortality rates. We also did not have specific information on young tree or long-term tree mortality for our case study. However, we considered differences between the urban heterogeneous environment and management conditions according to land use classes of parks, streets and mixed residential or commercial use. We consequently assigned our classified individual tree species to these classes, the boundaries of which we took from digital OpenStreetMap data. We also applied a buffer zone of 10 m for streets to account for trees with high proximity. The highest annual mortality rate of 3.5% was assigned to street trees due to multiple stress factors, such as pollution, sealed surface, traffic safety issues and other growth-limiting conditions. Recent studies showed similar mortality rates between 3.5 and 5.1% for urban street trees [109]. A tree mortality of 1% was set for parks, which we assumed to have fewer stress factors than street trees. We assigned a tree mortality of 2% to mixed areas because the space was likely to be limited for large tree growth. Although our mortality rates were based on the assumption of high uncertainty, results could be used to generally reflect a sensitivity concerning a mortality range of 1–3.5%. We achieved the mortality rates of our land use class by applying an annual stratified random selection of individual trees in our tree database. We did not exclude very old trees using a threshold of a maximum age, but we indicated high uncertainty when the average age of the tree population exceeded 80 years, which is unlikely for the majority of urban trees [110]. Our results summarized the dead biomass for each land use class, which was accumulated at 10-year intervals.

Planting: we applied a separate calculation of a potential tree planting initiative of 100,000 trees with a growth period of 70 years. Tree growth and alive biomass were calculated using a mixture of dominant tree species (Class mix, Table 1). We indicated the tree population half-life in our results—the years until 50% of the tree population is dead—for an annual static mortality rate of 3.5% (20 years), 2% (35 years) and 1% (69 years), and discussed how far an extensive replanting could compensate for alive biomass loss concerning our LCA of 60 years.

Miscellaneous: due to limitations of this study our calculations did not account for emissions of maintenance, tree production, planting, irrigation, processing of dead biomass, tree removal and residual biomass from regular pruning. We added the consequences of these limitations to our discussion.

#### Case results and discussion—urban forest carbon offset for streets, mixed and park areas

**Fig. 2:**
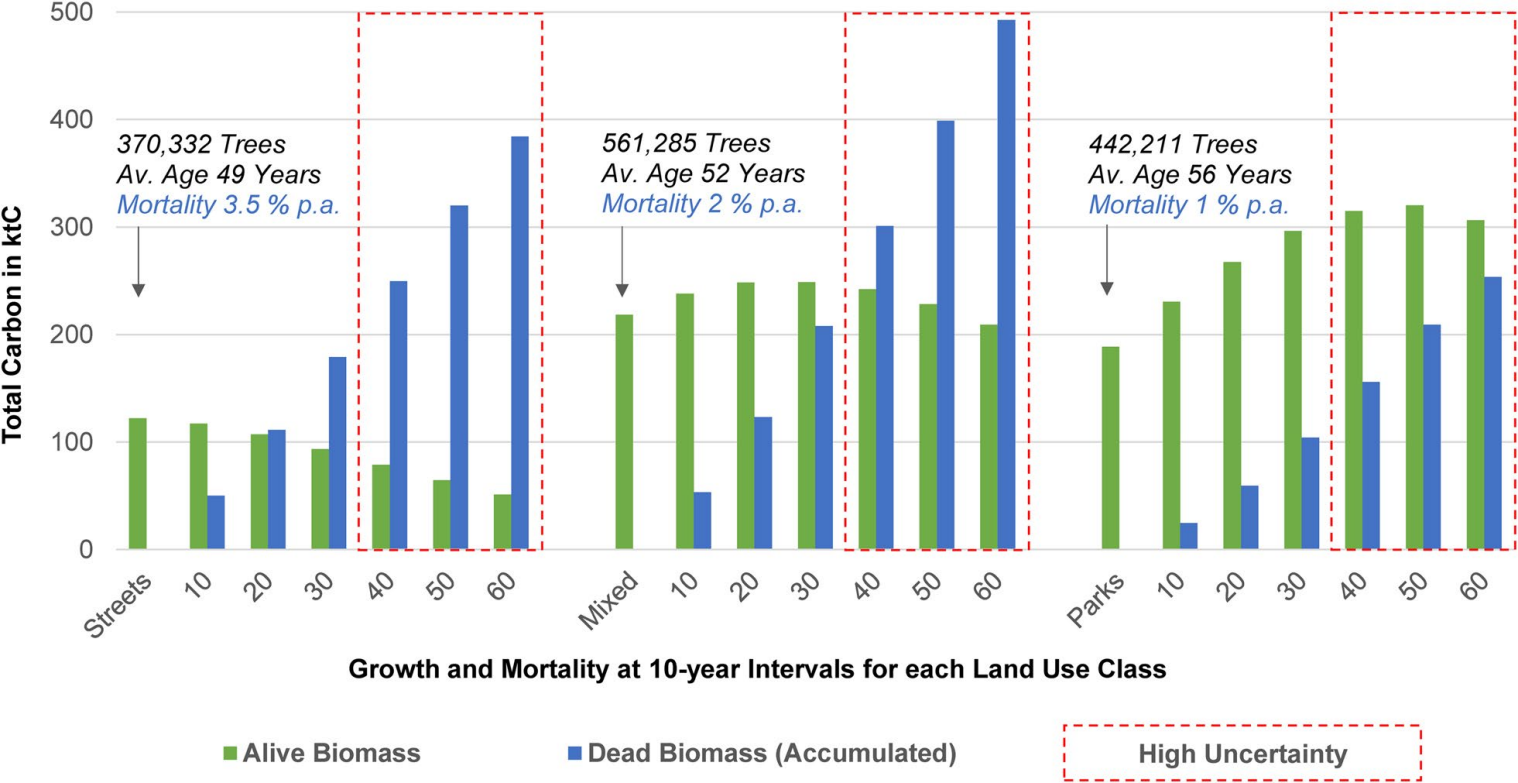
LCA of 60 years on urban forest carbon offset in Berlin. The carbon weight for alive and accumulated dead biomass is presented in kilotons (ktC) for our land use classes (streets, mixed, parks). The absolute tree population, the assumed mortality rate of each land use class and its average age is indicated for the LCA start in 2008. A red border marks a tree population of an unlikely very high average age (> 80 years)

Our LCA of 60 years on urban forest carbon offset clearly indicates the dominant impact of an increasing annual tree morality between our land use classes of parks (low, 1%), mixed areas (moderate, 2%) and streets (high, 3.5%) (Fig. 2). Street trees show a continuous decline in absolute alive biomass. After 20 years the amount of accumulated dead biomass is already higher than alive biomass. Trees of mixed areas reveal a slight increase in alive biomass, which levels off after 30 years and then continuously declines. Accumulated dead biomass of mixed areas exceeds alive biomass after 40 years. Park trees show a dominant effect of growth with an increase in alive biomass for 50 years and with a marginal decrease for the last 10 years of our assumed 60-year LCA.

The assumed low mortality for park trees leads to consistently larger quantities of alive than accumulated dead biomass. The average age of our total tree population was approximately 52 years. There were no extreme differences in the age structure between our land use classes. However, older trees are more likely to be found in parks, which have an average age of 56 years. Street trees have the youngest average age of 49 years and trees of mixed areas; 52 years. Our land use classes have a similar age standard deviation (streets: 15 years; mixed: 17 years; parks: 15 years). In this context, the average age structure for parks (high), mixed areas (moderate) and streets (low) corresponds to our assumed mortality differences between land use classes: low for parks, moderate for mixed areas and high for streets. Our land use classes exceed the average age of 80 years after 30 years of our LCA.

The carbon estimates per unit of land cover are consolidated for all land use classes at 10-year intervals in Table 2. We found that the carbon density of alive biomass in Berlin increases by almost 25% for the first 30 years of our LCA (2008–2038); thereafter, it continuously decreases. Our study concludes that the carbon density of alive biomass is always higher than the initial year of 2008 (7.3 tC/ha) if the average age of the total tree population is allowed to exceed 80 years. The initial sequestration rate decreases rapidly for the first 30 years. After this time period, the growth of the remaining tree population cannot compensate for its previous losses and the total alive biomass declines. The carbon density of accumulated dead biomass exceeds alive biomass density at 40 years of our LCA. At the end of our LCA (2068), the carbon density of dead biomass (16.2 tC/ha) is more than 100% higher than alive biomass (8.0 tC/ha).

**Table 2:** Temporal development of urban forest carbon estimates in Berlin

Carbon estimates	LCA						
Land cover of 700 km^2^	Start	10	20	30	40	50	60
Alive biomass							
Density average (tC/ha)	7.3	8.3	8.9	9.1	9.0	8.7	8.0
Dead biomass (accumulated)							
Density average (tC/ha)		1.8	4.2	7.0	10.0	13.3	16.2

**Fig. 3:**
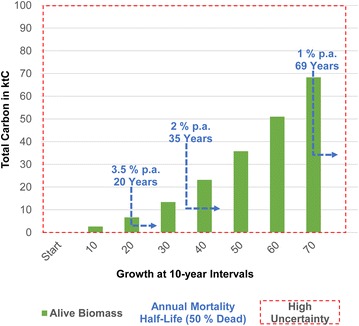
Potential tree planting initiative of 100,000 trees. Calculations of alive biomass were based on a mixture of dominant tree species in Berlin (Class mix, Table 1) with a 70-year growth period. The carbon weight is presented in kilotons (ktC). Tree population half-life is shown for high, moderate and low annual mortality. High uncertainty (red border) is generally assumed due to lack of knowledge concerning factors such as young tree mortality and other natural and anthropogenic disturbances

Results of our potential tree planting of 100,000 trees of mixed dominant tree species (Table 1) show an increase of alive biomass with a total of approximately 68 ktC for 70 years of growth (Fig. 3). The planted tree population decreases to 50% (population half-life), resulting from a high annual mortality (3.5%) with an alive biomass of approximately 3.5 ktC after 20 years of growth. A moderate mortality (2%) leads to an alive biomass of approximately 10 ktC after 35 years of growth and approximately 35 ktC after 69 years of growth for a low mortality (1%). Street trees continuously lose alive biomass (Fig. 2): 15.2 ktC in the first 20 years of our LCA. Our assumed extensive tree planting of 100,000 trees (+ ca. 3.5 ktC at population half-life of 20 years) needs to be more than four times larger to compensate for this short-term loss of alive biomass. Concerning estimates of mixed areas, it takes 40 years till carbon sequestration cannot compensate our assumed tree loss of 2% p.a. any longer. The relevant decrease of alive biomass is less than 1 ktC (Fig. 2). Therefore, planting 100,000 trees in mixed areas (+ 10 ktC at population half-life of 35 years) is likely to compensate for the loss of alive biomass. We assumed a low mortality rate of 1% p.a. for park trees, which leads to an extended time span of 60 years till carbon sequestration cannot compensate tree loss any longer. The relevant decrease of alive biomass is 14.1 ktC (Fig. 2). Consequently, a planting initiative of 100,000 park trees (+ 35 ktC at population half-life of 69 years) is likely to extend the amount of alive biomass for long-term purposes.

In this case study, we could make use of recent progress in high resolution remote sensing to increase consistency of an LCA inventory on urban forest carbon offset. We did this by providing area-wide details of remotely sensed individual tree species. To the best of our knowledge, this is the first urban study combining individual tree metrics with spatially explicit species information for an LCA of urban forest carbon offset. Our results point out the challenges of assumed tree growth and mortality of street trees, mixed areas and parks, which greatly affect the future role of urban forest carbon offset. Furthermore, our findings call attention to the potential of tree planting to compensate for losses of alive biomass.

Our results show that urban forest carbon offset is dominated by our assumed tree mortality. The significant impact of an increasing mortality rate on urban forest carbon offset was also confirmed by Strohbach et al. [32]. Only park trees have a positive balance of alive biomass minus accumulated dead biomass for a 60-year LCA. In particular, the high loss of street and mixed trees reduced the overall growth potential of the remaining tree population. The increasing average age structure is also likely to augment the risk of tree loss. Therefore, our results certainly underline the necessity of adequate forest management applications across the city. It remains unclear, in how far the high amount of dead biomass contributes to net carbon offset, which grew twice as much as alive biomass after 60 years (Table 2). Other LCA approaches utilized advanced processing of dead biomass, such as composting or wood chip production for energy consumption [30, 32]. Consequently, the decomposition of dead biomass means a release of carbon emissions, and urban forests of our case study would be a net carbon source. However, our case study can become a net carbon sink if dead biomass is used for energetic purposes and substitutes fossil energy.

Our results show the possibilities of extensive tree planting. Replanting 100,000 trees has the capability to compensate for losses in alive biomass. However, this potential requires rapid replanting, low mortality rates (< 2% p.a.) as well as extended tree longevity exceeding 80 years. The lack of relevant data on future tree replanting rates, mortality, growth and longevity make it difficult to provide long-term prognoses for our case study. Increasing disturbances in growth, mortality and replanting rates would affect the spatial expanse and quantity of alive biomass. Today’s financial constraints of cities might limit investments into green infrastructure, such as an extensive tree replanting program [111]. In particular, the lack of young tree mortality data causes high uncertainty concerning the true quantitative requirements to compensate for tree loss [112]. As such, further discussion is needed on urban forest management plans and political decision-making concerning urban dwellers’ desires and climate change adaptation in particular [113]. In this context, our city-wide average of carbon density in Berlin most likely falls in the lower range of urban forest studies obtained from globally selected cities of temperate climate zones (11–38 tC/ha) [17]. The temporal development of our carbon density shows that estimates will remain in the lower range if management plans do not consider extensive replanting and densification.

It should be made clear that, in reality, our assumed values for tree growth, mortality and planting would be an exception rather than the rule. These values can vary widely and be extremely site- and species-specific [107, 114]. Climate change especially could increase stresses on urban forests and change their value, which would then require adapted management strategies [113, 115]. Our assumed constant mortality rates did not describe the complexity of natural and anthropogenic disturbances adequately [110]. However, the lack of mortality relevant statistics and in situ data from Berlin did not allow us to further calibrate or validate our case study. Moreover, the high uncertainty of our case study results refers to the high average age of the tree population, which had already reached an average of 80 years after 30 years of our LCA. Therefore, our results should be taken carefully and one should consider the possibility of an extensive underestimation of dead biomass for the period from 2038 till 2068. In this regard, only few studies exist on urban tree growth and longevity and require further improvements in quantitative assessments [116]. Due to limitations we did not consider pruning trees, which would increase the amount of dead biomass for our estimates. Pruning of 10% above-ground biomass at 10-year intervals could be considered a common practice according to recent studies [30, 32]. Our simplified case study also excluded emissions related to tree planting, such as maintenance, tree production, transportation, planting, irrigation, processing of dead biomass and tree removal. These might be considered less relevant for the net carbon offset of our case study because recent studies on LCA of urban tree planting showed a relative small share on emissions of 1–5% of the total tree biomass (alive plus dead) [30, 32]. However, an accounting for decomposition of dead biomass, dead roots and irrigation can significantly increase the share of carbon emissions.

In general, our LCA inventory data of remotely sensed individual tree species contributed consistent details across large areas, and therefore avoided current disadvantages of general field data-based methods or requirements of widely used approaches of the i-Tree ECO model. Zhao et al. [29] confirmed the advantages of LiDAR data and individual tree detection to retrieve consistent and area-wide LCA inventory data, which our case study could increase in precision by providing additional tree species information. However, Zhao’s and our LiDAR-based remote sensing approach did not include understory and very small trees (< 3 m) [15], which can grow to a considerable quantity in the long term. Further differentiation for our tree species (Table 1) is also a suggestion for improvement because our mixed class covered a considerable fraction (> 25%) of the total tree population. This can be of utmost importance when considering the role of invasive species. For instance, Horn et al. [117] pointed out the role of invasive species on urban forest carbon offset, which was approximately 5% for a subtropical urban forest. Even more relevant were temporal changes of the urban species structure. Nowak et al. [118] showed slight changes in tree canopy size from 1999 to 2009, in Syracuse, New York; however, the number of trees increased by more than 20% with invasive species as the main cause. This indicates the importance of the individual tree level and changes in forest composition. Therefore, high resolution remote sensing has great potential to monitor necessary environmental details and should considerably improve tree species classification and individual tree detection in urban settings.

We can conclude that our simplified LCA of urban forest carbon offset offers new insights into how high resolution remote sensing can be used as a consistent baseline of individual tree species. Future progress in remote sensing will show if we can extend the classification of tree species and the detection of individual trees with increasing precision. This might change the way we assess major knowledge gaps regarding the role of invasive tree species and private properties in particular. Time series of remotely sensed data can offer the potential to validate and calibrate predictive models due to changes in tree size, species structure, age structure and replanting. This is particularly true for the current lack of urban tree mortality data, where remote sensing can help to detect potential risks and stresses and reveal irregular or chaotic spatiotemporal patterns of disturbances. All of this can help cities and other stakeholders to consider the role of urban forest carbon offset more adequately; for instance, to complement current carbon balances.

### Reducing uncertainties part 2—remotely sensed long-term monitoring for considering urban forest dynamics

Remotely sensed high resolution monitoring would certainly contribute to improving an LCA of urban forest carbon offset by providing additional data for calibration and validation. Researchers and professional practitioners have requested long-term ecological studies to extend the knowledge of urban forest dynamics. However, few studies on urban tree growth and related changes exist, and they all rely on regional networks or research related projects, such as the International Tree Failure Database (ITFD), for instance. Experts are aware of remote sensing’s opportunities for developing a more consistent urban forest database, but it is still far from being realized as a common international standard [116].

#### Tree growth and pruning

Diameter at breast height (DBH) has commonly been used for tree growth analysis [119, 120]. Nonlinear and linear regression models have successfully shown accurate estimates of DBH using crown width or a combination with tree height [54, 121]. Performing ITD with high resolution remote sensed data is appropriate for predicting crown width and tree height, but the variability of the resulting estimates can be high. For example, additional age to DBH-growth functions allow for estimating the urban forest age structure, but available studies rarely address the various urban tree species, tree dimensions and site conditions. Additionally, increasing climate change effects can cause water and heat stress, substantially reducing tree growth [114, 122–124]. Therefore, the variability of remotely sensed tree dimensions, as mentioned earlier in this review, does not allow for deriving the specific age of a single tree. We rather suggest providing a certain range of the tree age, which can be used as a baseline for additional processing or modeling.

Future studies and applications should place greater focus on urban forest dynamics using high resolution remote sensing. Detailed characterizations and monitoring highly benefit from increasing temporal resolution in particular. This allows for potential change detection of urban tree growth and pruning and for monitoring abrupt changes like replanting or tree loss. However, precise estimates would come at a high price, as change detection errors are likely to significantly increase as a function of heterogeneity, quality and resolution of imagery, or misregistration errors of remotely sensed time series [125]. This makes it difficult to reliably estimate changes over time for an individual tree. Regarding precise estimates of urban forests, little is known on monitoring the changes of individual trees using very high resolution remote sensing, such as Ardila et al. [41] successfully used a region-based active contours approach for a time series of multispectral data to detect abrupt and gradual changes of individual trees. For example, remotely sensed tree dimensions would allow for improved monitoring of residual biomass, large quantities of which can be obtained from urban forests for energy purposes [126]. Additionally, Sajdak et al. [127] applied predictive modeling approaches and showed correlations between residual biomass and the parameters of crown diameter or stem DBH, which could be derived from remotely sensed individual trees. Due to the variability of remotely sensed tree dimensions, we suggest stretching the intervals for monitoring urban forests in order to retrieve notable physical changes of tree growth and derived parameters. For this very reason, further research is necessary to confirm the precision of gradual changes, like tree growth (height and crown) and pruning, using high temporal resolution remote sensing.

#### Mortality and replanting

Temporal increases and decreases of the urban forest canopy is crucial information for an LCA of urban forest carbon offset. Until now, few studies have addressed urban forest dynamics across large areas, and most of them use moderate resolution (30 m pixel size) and adapt ground-based carbon measures to vegetation indices using spectral bands of Landsat satellite data [128, 129]. Moderate resolution of Landsat satellite imagery is likely to underestimate urban forest cover. However, Landsat satellite time series was sufficient for indicating a long-term trend of urban forest increase (growth + replanting) and decrease (mortality), which was likely to be balanced for urban forests in Syracuse, US from 1985 to 1999 [128], or to have highly increased for urban forests and shrubs in Xi’an, China from 2004 to 2010 [129]. The importance of temporal changes has also made remote sensing a source for validating reported facts. Nowak et al. [130] chose higher resolution time series to better address the trend of urban tree cover across the US. They manually digitized high resolution paired aerial photographs and Google Earth imagery (selection of 2001–2009 for 20 US cities and 1000 randomly selected urbanized areas). The results of this sampling approach indicated a decline of forest cover for most US cities at a rate of approximately 7900 ha/year, or 4 million trees per year. Other work of Merry et al. [131] indicated only slightly changes in tree canopy size for longer periods of time for selected US cities (1951–2010, Detroit and Atlanta). However, they suggested advanced remote sensing techniques to consider variations in spatial distribution more adequately. For example, the tree canopy cover report of Boston, US showed significant overestimation due to misclassification of grass and shrubs as tree canopy compared to an assessment performed with high resolution QuickBird satellite and LiDAR data [47].

Due to the increasing availability of high resolution three-dimensional remote sensing data, the individual tree level is the most promising level for future applications of urban forest dynamics [72]. Multiple reasons of mortality and replanting have remained unresolved as of yet, but a time series of individual trees across large areas would provide spatially explicit information, which is rarely available. This would at least contribute to better understanding urban forest dynamics, such as hot spots of mortality, species and site-specific differences, for example. Multitemporal LiDAR is of growing interest for better understanding and monitoring spatiotemporal dynamics of forest carbon [132–135]. To the authors’ knowledge, multitemporal LiDAR has not yet been applied to changes of urban forest carbon. However, due to a lack of resources a precise timing of acquiring a single image at the growing season (leaf development) might be considered advantageous for detecting dead trees (no leaf development). Additionally, tree health issues, which, for instance, correlate to multispectral information of remote sensing data, should be used to indicate areas of potential risk [136]. This can advance an LCA to better consider the effects of temporal urban forests’ stresses.

## Conclusions

Recent progress in high resolution remote sensing and methods is adequate for delivering more precise details on the urban tree canopy, individual tree metrics, species and age structures compared to conventional land use/cover class approaches. These area-wide consistent details can update life cycle inventories for more precise future prognoses.

Additional improvements in classification accuracy can be achieved by a higher number of features derived from remote sensing data of increasing resolution, but first studies on this subject indicated that a smart selection of features already provides sufficient data that avoids redundancies and enables more efficient data processing. Automated and efficient processing of very high resolution data should be employed if possible due to the increasing workload, variability of data and user interaction, which can cause unresolved uncertainties. More consistent reporting of uncertainties and a better understanding of today’s locally tailored approaches would allow for more generic and transferable approaches. However, this will not neglect today’s advantages of high resolution remote sensing, which already extend the possibilities of traditional field-based retrieval of heterogeneous urban forest structures across large and private areas and should be applied more frequently.

In the matter of temporal changes and reliable estimates, more attention is required to detect the changes of gradual growth, pruning and abrupt changes to the planting and mortality of individual trees. Therefore, precise long-term ecological monitoring of urban forest dynamics should be intensified, especially due to increasing climate change effects. The results would be beneficial for calibrating and validating recent studies of urban forest carbon offset, which have so far focused on the status quo and net sequestration for the following year but have scarcely addressed a longer timeframe. Furthermore, precise change detection is highly relevant for the supply and demand of urban forest carbon offset.

A precise global estimate of urban forest carbon offset is still missing. However, upscaling approaches have improved national estimates in the US and Canada using higher resolution remote sensing data, which should be continued to reach an initial global coverage. The future role of urban forest carbon offset can be made more relevant if more standardized assessments are made available for science and professional practitioners, and the ever increasing availability of high resolution remote sensing data and the progress in data processing allows for precisely that.

## Abbreviations

AVHRR: advanced very high resolution radiometer
C: carbon
CHM: canopy height models
CO_2_: carbon dioxide
DBH: diameter at breast height
EPA: Environmental Protection Agency
FIA: Forest Inventory and Analysis
ha: hectare
IPCC: Intergovernmental Panel on Climate Change
ISO: International Organization for Standardization
ITD: individual tree detection
ITFD: International Tree Failure Database
LCA: life cycle assessment
LiDAR: light detection and ranging
NBCD: National Biomass and Carbon Dataset
NDVI: normalized difference vegetation index
RMSE: root means square error
t: ton
TM: thematic mapper
UAV: unmanned aerial vehicles
USDA: United States Department of Agriculture
